# Construction on of a Ferroptosis-Related lncRNA-Based Model to Improve the Prognostic Evaluation of Gastric Cancer Patients Based on Bioinformatics

**DOI:** 10.3389/fgene.2021.739470

**Published:** 2021-08-23

**Authors:** Jiahui Pan, Xinyue Zhang, Xuedong Fang, Zhuoyuan Xin

**Affiliations:** ^1^The Key Laboratory of Zoonosis Research, Chinese Ministry of Education, College of Basic Medical Science, Jilin University, Changchun, China; ^2^Department of Gastrointestinal Colorectal and Anal Surgery, China-Japan Union Hospital of Jilin University, Changchun, China

**Keywords:** ferroptosis, lncRNAs, prognostic model, gastric cancer, bioinformatics and computational biology

## Abstract

**Background:**

Gastric cancer is one of the most serious gastrointestinal malignancies with bad prognosis. Ferroptosis is an iron-dependent form of programmed cell death, which may affect the prognosis of gastric cancer patients. Long non-coding RNAs (lncRNAs) can affect the prognosis of cancer through regulating the ferroptosis process, which could be potential overall survival (OS) prediction factors for gastric cancer.

**Methods:**

Ferroptosis-related lncRNA expression profiles and the clinicopathological and OS information were collected from The Cancer Genome Atlas (TCGA) and the FerrDb database. The differentially expressed ferroptosis-related lncRNAs were screened with the DESeq2 method. Through co-expression analysis and functional annotation, we then identified the associations between ferroptosis-related lncRNAs and the OS rates for gastric cancer patients. Using Cox regression analysis with the least absolute shrinkage and selection operator (LASSO) algorithm, we constructed a prognostic model based on 17 ferroptosis-related lncRNAs. We also evaluated the prognostic power of this model using Kaplan–Meier (K-M) survival curve analysis, receiver operating characteristic (ROC) curve analysis, and decision curve analysis (DCA).

**Results:**

A ferroptosis-related “lncRNA–mRNA” co-expression network was constructed. Functional annotation revealed that the FOXO and HIF-1 signaling pathways were dysregulated, which might control the prognosis of gastric cancer patients. Then, a ferroptosis-related gastric cancer prognostic signature model including 17 lncRNAs was constructed. Based on the RiskScore calculated using this model, the patients were divided into a High-Risk group and a low-risk group. The K-M survival curve analysis revealed that the higher the RiskScore, the worse is the obtained prognosis. The ROC curve analysis showed that the area under the ROC curve (AUC) of our model is 0.751, which was better than those of other published models. The multivariate Cox regression analysis results showed that the lncRNA signature is an independent risk factor for the OS rates. Finally, using nomogram and DCA, we also observed a preferable clinical practicality potential for prognosis prediction of gastric cancer patients.

**Conclusion:**

Our prognostic signature model based on 17 ferroptosis-related lncRNAs may improve the overall survival prediction in gastric cancer.

## Introduction

Gastric cancer is one of the most serious gastrointestinal malignant tumors worldwide, which is the third most common cause of cancer-related death. Resulting from unhealthy lifestyle habits, *Helicobacter pylori* infection, and an increasing social burden, 1,089,103 new cases occurred in 2020, while deaths reached 768,793 cases (Sung et al., 2021). Gastric cancer has contributed to a massive burden for both sexes in China (Feng et al., 2019; Cao et al., 2021). Despite surgical and adjuvant therapy technologies having improved rapidly, the overall survival (OS) rates for gastric cancer patients still remain very poor. Especially for patients suffering advanced gastric cancer, the 5-year survival rate is lower than 20% (Ferlay et al., 2019; Tan, 2019). Nowadays, the TNM classification of malignant tumors is still the globally recognized standard for classifying the extent of spread of gastric cancer (Brierley et al., 2016). However, the responses to treatment and individual differences may affect the prognostic evaluation of patients with the same TNM classification status due to indefinite genetic features. Therefore, novel prognostic markers are urgently needed for gastric cancer.

Ferroptosis is an iron-dependent form of programmed cell death (PCD) (Dixon et al., 2012; Tang et al., 2019) that may regulate cell death by mainly relying on the accumulation of reactive oxygen species (ROS), resulting in the dysregulation of glutathione peroxidase activities (Li et al., 2020). Recent studies have shown that aberrant iron metabolism is an important risk factor that may affect the initiation and progression of cancer, which would control the prognosis of patients (Xie et al., 2016; Roemhild et al., 2021). Long non-coding RNAs (lncRNAs) are endogenic functional RNAs that can regulate the activities of cancer-related metabolic pathways (Ransohoff et al., 2018). lncRNAs have been confirmed to be able to regulate the ferroptosis of cancer cells as epigenetic and metabolic regulators (Mao et al., 2018; Wang et al., 2019; Wu et al., 2020). What is more is that their higher molecular stability makes lncRNAs a better form of biomarker (Statello et al., 2021).

In this study, we collected the transcriptome and clinicopathological and OS information for gastric cancer from The Cancer Genome Atlas (TCGA) database. We also collected 259 ferroptosis-related genes from FerrDb. Using the DESeq2 method, we identified the differentially expressed (DE) lncRNAs and ferroptosis-related genes. Then, we constructed the ferroptosis-related “lncRNA–mRNA” co-expression network. Functional annotation analysis has shown that these lncRNAs would control the metal ion and ROS response, which may affect the OS rates of gastric cancer patients. Based on these ferroptosis-related DE-lncRNAs, we then constructed a model for gastric cancer prognostic evaluation based on 17 ferroptosis-related lncRNAs. The area under the receiver operating characteristic (ROC) curve (AUC) value for this model is 0.751, which is better than those of other published models. The nomogram and the decision curve analysis (DCA) results showed a preferable clinical practicality potential for this model. Therefore, the prognostic evaluation model constructed in our study may improve prognosis prediction for gastric cancer patients.

## Materials and Methods

### Data Collection

From TCGA database^1^ (Hutter and Zenklusen, 2018), we collected the HTSeq-Count data, clinicopathological data, and OS information for stomach adenocarcinoma (STAD). In total, we have downloaded the transcriptome data of 434 tissues (387 STAD tissues and 47 normal tissues from 387 patient samples) and collected the clinicopathological features including age, gender, TNM stage, clinical stage, and grade stage. Then, we also download TCGA gene annotation information version 22 from the GENCODE database^2^ (Frankish et al., 2019). The 259 ferroptosis-related genes were collected from the FerrDb database^3^ (Zhou and Bao, 2020). In addition, we downloaded the annotated gene sets for gene set enrichment analysis (GSEA) from MSigDB^4^ (Subramanian et al., 2005).

### Identification of Ferroptosis-Related DE-lncRNAs

Based on R (ver. 4.0.3), we used the “DESeq2” package to screen the DE lncRNAs and genes, with the threshold values | log2(FoldChange)| > 1 and *P* < 0.05. The read count data were used for the DESeq2 workflow, and the data normalization processes were integrated into the DESeq2 workflow. Subsequently, through Pearson’s correlation analysis, 1,062 ferroptosis-related DE-lncRNAs were identified, with the threshold values | cor| ≥ 0.4 and *P* < 0.05.

### Visualization of the Ferroptosis-Related lncRNA–mRNA Co-expression and Protein–Protein Interaction Networks

Based on Pearson’s correlation, we visualized the ferroptosis-related lncRNA--mRNA co-expression network using Cytoscape (ver. 3.8.2). Next, protein--protein interactions (PPIs) were identified using the STRING dataset^5^ (Szklarczyk et al., 2019).

### Functional Enrichment Analysis

Based on R (ver. 4.0.3), we used the “clusterProfiler” package to perform Gene Ontology (GO) annotation and Kyoto Encyclopedia of Genes and Genomes (KEGG) pathway enrichment analysis, with default parameters. Afterward, we performed GSEA using the gene set “msigdb.v7.4.entrez.gmt” download from MSigDB.

### Construction of the Ferroptosis-Related lncRNA-Based Prognostic Model

Based on R (ver. 4.0.3), we used the “survival” and “glmnet” packages to perform Cox regression with the least absolute shrinkage and selection operator (LASSO) algorithm and multivariate Cox regression analysis. We used the identified 1,062 DE ferroptosis-related lncRNAs to construct the gastric cancer prognostic model. Then, utilizing the prognostic model, we calculated the RiskScore for each patient samples with the formula: RiskScore = $\sum_{1}^{\text{n}}{\text{Cor}}_{\text{lncRNAi}}\times {\text{Exp}}_{\text{lncRNAi}}$ [where Cor_lncRNAi_ represents the correlation coefficient of interfering lncRNAi (lncRNAi), Exp_lncRNAi_ represents the expression level for lncRNAi, and *n* represents the number of lncRNA signatures]. Based on the median RiskScore, the patients were divided into a High-Risk group (over the median RiskScore) and a Low-Risk group (no more than the median RiskScore).

### Nomogram

Based on R (ver. 4.0.3), we used the “rms” package to construct the nomogram in order to predict the 1-, 3-, and 5-year OS rates of gastric cancer patients. We also constructed the calibration curve and evaluated the consistency between the OS rates predicted by the nomogram and the actually observed OS rates. Using the “rmda” package, we performed the DCA to compare the Net-Benefits with the different predictors.

### Survival and ROC Analysis

We used the “survival” and “survminer” packages with R (ver. 4.0.3) to perform the Kaplan–Meier (K-M) survival curve analysis on the OS rates. Then, the “survivalROC” package was also used for ROC analysis. The AUC value was calculated for the evaluation of the prediction accuracy of the ferroptosis-related lncRNA-based prognostic model.

## Results

### Identification of Ferroptosis-Related DE-lncRNAs

**FIGURE 1:**
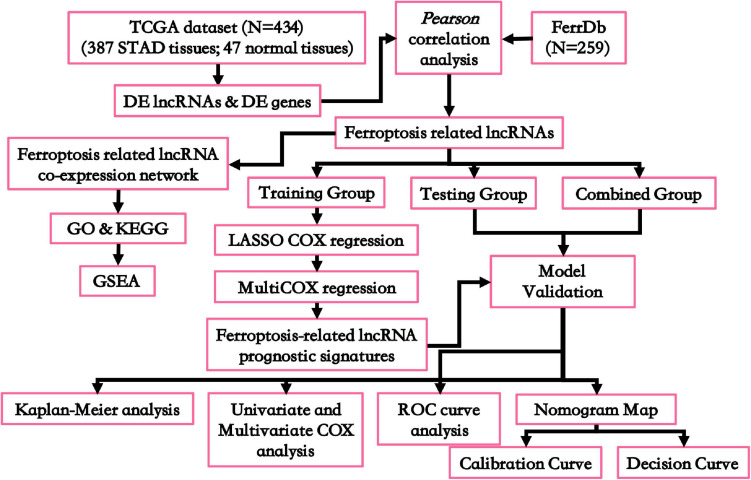
Flowchart for this work. DE, differentially expressed; GO, Gene Oncology; KEGG, Kyoto Encyclopedia of Genes and Genomes; GSEA, gene set enrichment analysis; ROC curve, receiver operating characteristic curve.

Figure 1 shows the workflow for this study. To identify the ferroptosis-related DE-lncRNAs among gastric cancer tissues, we firstly collected the transcriptome data of 434 tissues (387 STAD tissues and 47 normal tissues from 387 patient samples) from TCGA database. Based on the integrality of the information on OS, a total of 370 patient samples were included for subsequent analysis. Afterward, the patients were divided randomly into a training group (containing 270 patient samples) and a testing group (containing 100 patient samples). From the FerrDb database (Zhou and Bao, 2020), 259 ferroptosis-related genes were collected simultaneously (Supplementary Table 1).

Using the DESeq2 method, we have detected 4,466 DE genes (2,125 upregulated and 2,341 downregulated) (Supplementary Table 2) and 3,391 DE-lncRNAs (2,394 upregulated and 997 downregulated) (Supplementary Table 3). Next, we identified 59 ferroptosis-related genes that were aberrantly expressed (21 upregulated and 38 downregulated) (Supplementary Table 4). Through Pearson’s correlation analysis, we identified 1,062 ferroptosis-related DE-lncRNAs among the gastric cancer tissues (Supplementary Table 5).

### Functional Annotation for the Ferroptosis-Related lncRNA–mRNA Co-expression Network

**FIGURE 2:**
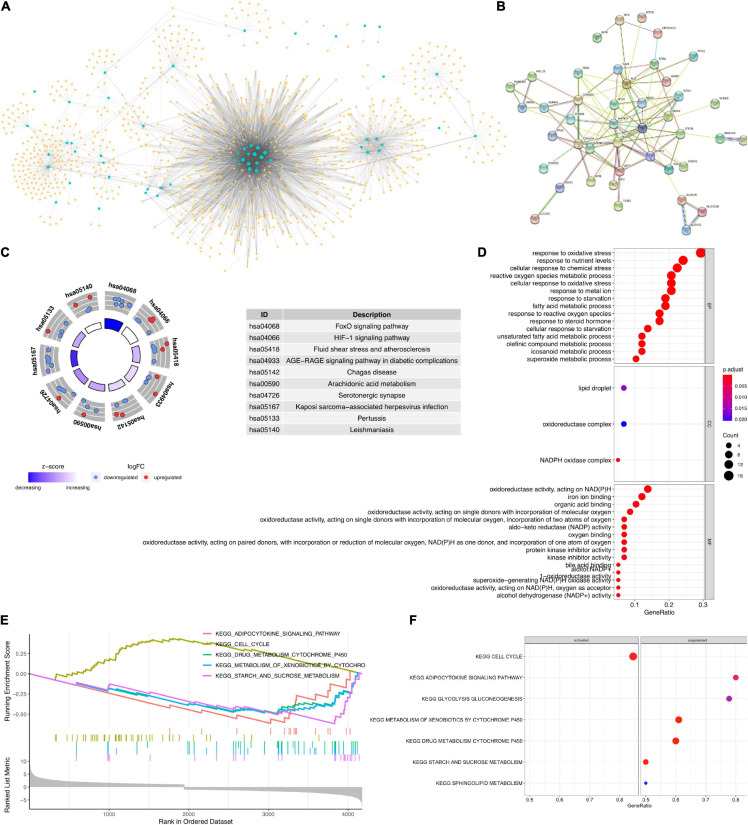
**(A)** Ferroptosis-related lncRNA–mRNA co-expression network. *Blue circles* are differentially expressed (DE) ferroptosis-related genes. *Yellow diamonds* are co-expressed lncRNAs. **(B)** Protein–protein interaction (PPI) network for the DE ferroptosis-related genes involved in the co-expression network. **(C)** Kyoto Encyclopedia of Genes and Genomes (KEGG) enrichment analysis for the targets of ferroptosis-related lncRNAs. **(D)** Gene Ontology (GO) annotation analysis for the targets of ferroptosis-related lncRNAs. **(E,F)** Gene set enrichment analysis.

To analyze the effects of the ferroptosis-related lncRNAs on gastric cancer prognosis, we constructed a ferroptosis-related lncRNA–mRNA co-expression network (Figure 2A, detailed in Supplementary Table 5). Among which, we also screened the PPIs (Figure 2B, detailed in Supplementary Table 6). Subsequently, KEGG and GO annotations were used to predict the potential biological functions for the ferroptosis-related lncRNAs. We identified the KEGG terms “hsa04068 FOXO signaling pathway” and “hsa04066 HIF-1 signaling pathway” as significantly enriched (Figure 2C, detailed in Supplementary Table 7). These results might indicate that, through regulating glucose metabolism, oxidative stress resistance, or cell cycle, ferroptosis-related lncRNAs would regulate the stress resistance abilities of gastric cancer cells, which would affect the OS rates indirectly. The GO annotation results also supported this inference. We identified that the GO biological process terms “GO:0006979 response to oxidative stress,” “GO:0042594 response to starvation,” “GO:0010038 response to metal ion,” and “GO:0062197 cellular response to chemical stress” were most significantly enriched (Figure 2D, detailed in Supplementary Table 8). Furthermore, GSEA was used to identify the ferroptosis-related lncRNA involved pathway enrichment features among gastric cancer tissues (Figure 2E). We identified the “cell cycle” pathway to be activated (Figure 2F). All the above results, together, indicated that the ferroptosis-related lncRNAs would control the prognostic status of gastric cancer patients, which would be potential features of gastric cancer prognosis prediction.

### Construction of Ferroptosis-Related Prognosis Prediction lncRNA Signatures

**FIGURE 3:**
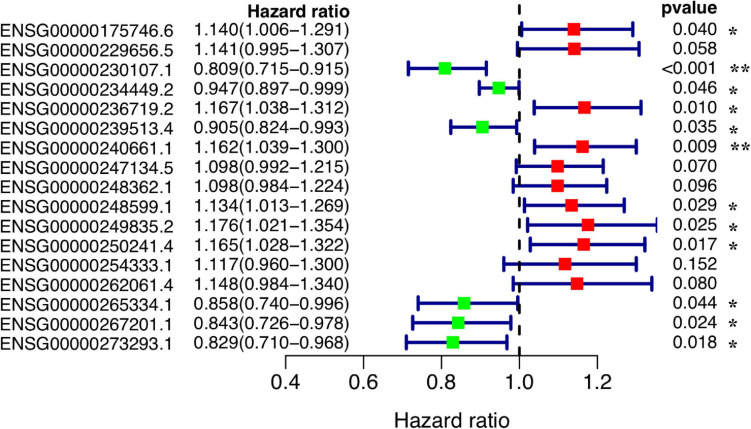
Forest plot of the prognostic values of the prognostic model based on 17 ferroptosis-related lncRNAs. **P < 0.01; *P < 0.05.

Based on the expression levels of the 1,062 DE ferroptosis-related lncRNAs and the OS information, we have built a gastric cancer prognostic model. Using univariate Cox regression analysis with the LASSO algorithm (Supplementary Figure 1) and multivariate Cox regression, we have constructed a prognostic model for gastric cancer patients based on 17 ferroptosis-related lncRNAs (Table 1). Among the 17 lncRNAs, 11 (namely, ENSG00000249835.2, ENSG0000023671 9.2, ENSG00000250241.4, ENSG00000240661.1, ENSG0000026 2061.4, ENSG00000229656.5, ENSG00000175746.6, ENSG00000 248599.1, ENSG00000254333.1, ENSG00000247134.5, and ENSG00000248362.1) were identified as prognostic risk factors, while the other six (namely, ENSG00000234449.2, ENSG0000023 9513.4, ENSG00000265334.1, ENSG00000267201.1, ENSG 00000273293.1, and ENSG00000230107.1) were identified as prognostic protective factors (Figure 3).

**TABLE 1:** Cox regression analysis with the LASSO algorithm for the prognostic model based on 17 ferroptosis-related lncRNAs.

**ID**	**Coefficient**	**HR**	**HR.95L**	**HR.95H**	***P*-value**
ENSG00000175746.6	0.13083909	1.13978436	1.00612336	1.2912019	0.03979336
ENSG00000229656.5	0.1314752	1.14050963	0.99540951	1.30676087	0.05826577
ENSG00000230107.1	–0.2118903	0.80905345	0.71506735	0.91539277	0.00077086
ENSG00000234449.2	–0.0546791	0.94678896	0.89737406	0.99892494	0.04557659
ENSG00000236719.2	0.15427449	1.16681112	1.03795681	1.31166169	0.00976813
ENSG00000239513.4	–0.1001319	0.90471807	0.82430676	0.99297351	0.03499291
ENSG00000240661.1	0.15010736	1.16195899	1.03876867	1.29975877	0.00866086
ENSG00000247134.5	0.09330239	1.09779365	0.99229873	1.21450411	0.0702977
ENSG00000248362.1	0.09304341	1.09750938	0.98375633	1.22441584	0.09559105
ENSG00000248599.1	0.12533296	1.13352581	1.01278503	1.2686609	0.02918059
ENSG00000249835.2	0.16191461	1.17575984	1.0208103	1.35422929	0.02472812
ENSG00000250241.4	0.1531115	1.16545492	1.02760484	1.32179716	0.01712812
ENSG00000254333.1	0.11093499	1.11732227	0.96006244	1.30034151	0.15175608
ENSG00000262061.4	0.13803103	1.14801117	0.98365591	1.33982791	0.07995866
ENSG00000265334.1	–0.15302	0.85811256	0.73955607	0.99567455	0.0436845
ENSG00000267201.1	–0.171106	0.84273222	0.72631476	0.97780966	0.02408258
ENSG00000273293.1	–0.1878586	0.82873189	0.70972024	0.96770039	0.01754597

*LASSO, least absolute shrinkage and selection operator; HR, hazard ratio; HR.95L, hazard ratio with lower 95% confidence index; HR.95H, hazard ratio with high 95% confidence index.*

### Prognostic Evaluation of the 17 Ferroptosis-Related lncRNA Signatures

**FIGURE 4:**
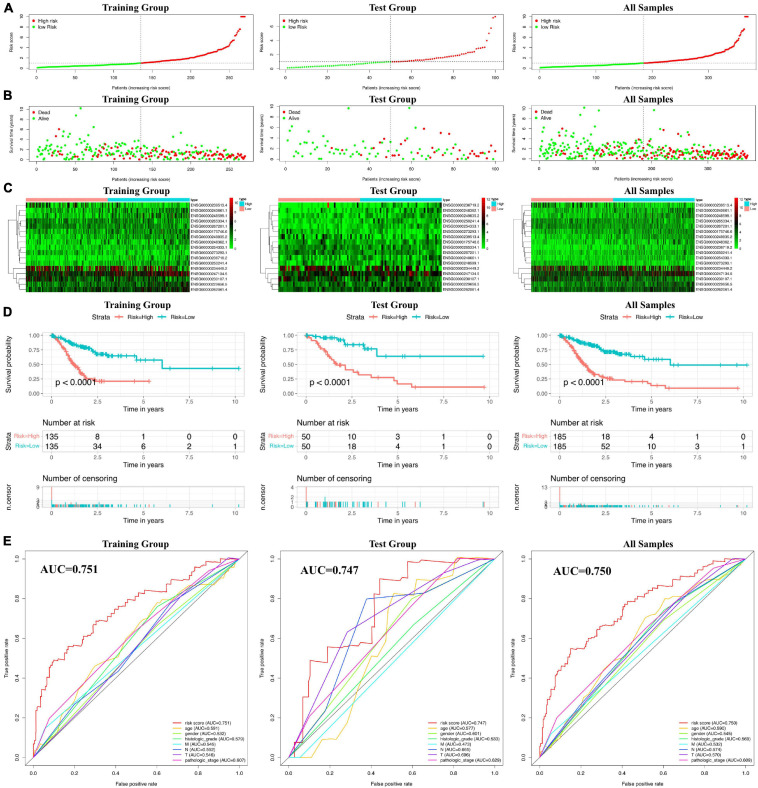
Prognostic risk scores calculated using the ferroptosis-related lncRNA-based prognostic model. **(A)** Distribuion of patients’ risk scores. **(B)** Patients’ overall survival (OS) status distribution. **(C)** Prognostic signature signal heatmaps. **(D)** Kaplan–Meier (K-M) survival curves for patients separated into the High-Risk and Low-Risk groups. **(E)** Receiver operating characteristic (ROC) curve analysis for verification of the prognostic value of the prognostic model based on 17 ferroptosis-related lncRNAs.

Using the prognostic model based on 17 ferroptosis-related lncRNAs, we calculated the RiskScore for each patient sample. Then, the patients were divided into a High-Risk group (RiskScore higher than the median RiskScore) and a Low-Risk group (RiskScore no more than the median RiskScore). From the RiskScore distribution dot plot, we found that more dead cases were observed in the High-Risk group (Figures 4A,B); the expressions of the 17 ferroptosis-related lncRNA signatures are exhibited as a heatmap in Figure 4C. Concurrently, the prognostic effectiveness of this model for gastric cancer patients’ OS status was evaluated using K-M survival curve analysis. We identified that the OS rates of the patients in the High-Risk group were significantly lower (*P* < 0.0001; Figure 4D). We also evaluated the prognostic accuracy of this model using ROC curve analysis. The results showed that the AUC value in the training group is 0.751 (Figure 4E). Additionally, we have verified the prognostic power and accuracy of the 17 ferroptosis-related lncRNA signatures between the test group and the combined group (containing all samples). The K-M survival analysis verified that the OS in the High-Risk group was significantly lower (*P* < 0.0001; Figure 4D). The AUC value in the test group is 0.747, while that in the combined group is 0.750 (Figure 4E). All these results, together, show that the 17 ferroptosis-related lncRNA signatures would be a valuable gastric cancer prognostic model.

### Correlation Between the 17 Ferroptosis-Related lncRNA Signature Model and the Clinicopathological Features

**FIGURE 5:**
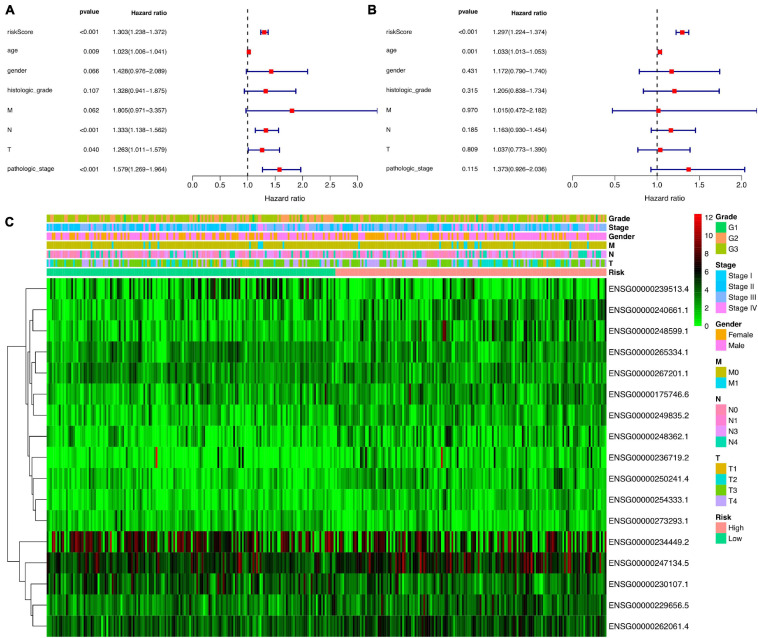
Cox regression analysis for the clinical phenotypes of gastric cancer patients. **(A)** Forest plot for the overall survival (OS) prognostic values through univariate Cox regression analysis. **(B)** Forest plot for the OS prognostic values through multivariate Cox regression analysis. **(C)** Relationship heatmap of the risk scores and clinical phenotypes of patients.

**FIGURE 6:**
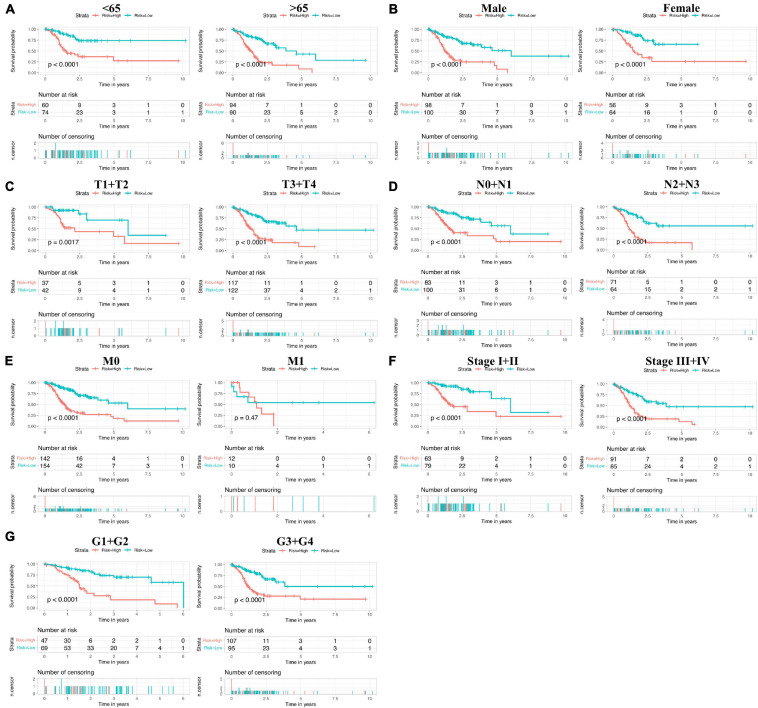
Subset group Kaplan–Meier (K-M) survival curves of patients. **(A)** Age <65 years (*left*) and ≥65 years (*right*). **(B)** Males (*left*) and females (*right*). **(C)** T1+T2 (*left*) and T3+T4 (*right*). **(D)** N0+N1 (*left*) and N2+N3 (*right*). **(E)** M0 (*left*) and M1 (*right*). **(F)** Stage I+II (*left*) and stage III+IV (*right*). **(G)** G1+G2 (*left*) and G3+G4 (*right*).

Next, to analyze whether the prognostic model based on 17 ferroptosis-related lncRNAs is an independent risk factor for gastric cancer prognosis, we performed Cox regression analysis. The results of the univariate COX regression in Figure 5A showed that the model (*P* < 0.001), N stage (*P* < 0.001), pathological stage (*P* < 0.001), age (*P* = 0.009), and T stage (*P* = 0.040) were meaningful for prognosis prediction. Concurrently, multivariate Cox regression showed that the model [*P* < 0.001, hazard ratio (HR) = 1.297, 95% CI = 1.224–1.374] and age (*P* = 0.001, HR = 1.033, 95% CI = 1.013–1.053) would be independent risk factors for the prognosis of gastric cancer (Figure 5B). In addition, the correlations between the prognostic model based on 17 ferroptosis-related lncRNAs and the clinicopathological features were exhibited as a heatmap (Figure 5C). We also performed the K-M survival analysis among each subset group that was separated according to the clinicopathological features. The results were shown as subset groups separated by age, gender, T stage, N stage, M stage, clinical stage, and grade (shown in Figures 6A–G, respectively). We observed that the OS rates in the High-Risk groups associated with age, gender, T stage, N stage, clinical stage, and grade were significantly lower.

### Construction and Evaluation of the Ferroptosis-Related lncRNA-Based Prognostic Nomogram

**FIGURE 7:**
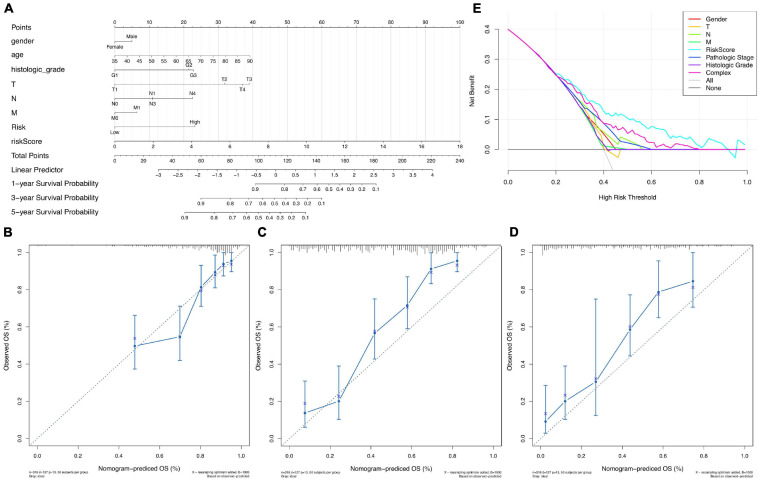
Nomogram of overall survival (OS) prediction of patients with gastric cancer. **(A)** Nomogram of both the prognostic model based on 17 ferroptosis-related lncRNAs and the clinical pathological factors. **(B–D)** Calibration curve analysis of the 1-year **(B)**, 3-year **(C)**, and 5-year **(D)** survival prediction accuracy of the nomogram. **(E)** Decision curve analysis (DCA) of the clinical practicality evaluation of the nomogram.

To evaluate the potential clinical practicality of the prognostic model based on 17 ferroptosis-related lncRNAs, we built a nomogram with the RiskScore and the clinicopathological features to predict the 1-, 3-, and 5-year OS rates of gastric cancer patients. As shown in Figure 7A, we observed that the higher the calculated RiskScore, the worse is the predicted prognosis. Then, we constructed the calibration curve to evaluate the consistency between the OS rates predicted by the nomogram and the actually observed OS rates. The results showed relatively good fits for the 1-, 3-, and 5-year OS prediction (Figures 7B–D, respectively). Finally, the DCA results (Figure 7E) demonstrated that this nomogram (the 17 ferroptosis-related lncRNA signature model) showed better clinical practicality for prognosis prediction of gastric cancer patients.

## Discussion

Ferroptosis is an iron-dependent cell death regulatory method that is distinct from apoptosis, necroptosis, and autophagy (Dixon et al., 2012). Ferroptosis is driven by the iron-dependent peroxidation of oxidizable membrane phospholipids (Doll et al., 2017). Recent studies have shown that the drug sorafenib, used to treat advanced hepatocellular carcinoma, exerts its anticancer activity mainly by regulating the ferroptosis process (Capelletti et al., 2020). TP53 could inhibit the ferroptosis process in human colorectal cancer cells by promoting the localization of DPP4 in the non-enzymatically active nucleus, and thus may promote the growth of human colorectal cancer cells (Xie et al., 2017). Apatinib would induce ferroptosis in gastric cancer cells through lipid peroxidation and then exerts its anticancer effect (Zhao et al., 2021). In this study, we have identified the DE ferroptosis-related lncRNAs among gastric cancer tissues. Through GO annotation analysis and KEGG pathway enrichment analysis, we found that the FOXO signaling pathway, the HIF-1 signaling pathway, and the cell cycle pathway were significantly enriched. FOXOs are essential transcription factors that play important roles in tumorigenesis because they have been shown to be dysregulated in many types of human cancers. Some studies have pointed out that the roles of FOXOs in breast cancer, colon cancer, and other cancers are contradictory. FOXO can induce cell death (Coomans, de Brachene and Demoulin, 2016). Besides, FOXO can enhance the proliferation, survival, and invasion abilities of cancer cells, which may promote the development of cancer (Storz et al., 2009; Sisci et al., 2013). Therefore, the FOXO signaling pathway may play an important role in a variety of cellular and physiological activities, such as cell proliferation, regulation of PCD, metabolism, and the disruption of the cell cycle (Ma et al., 2018). There are also some studies showing that the FOXO signaling pathway can be regulated to delay the progression of prostate and liver cancer (Shan et al., 2019; Li et al., 2021). The HIF-1 signaling pathway is involved in tumor growth, proliferation, and migration (Tian et al., 2015; Macklin et al., 2017). The HIF-1α signaling pathway mediates the promotion of CThrC1 in the migration and invasion of gastric cancer cells (Ding et al., 2020). The cell cycle is also important for the occurrence and development of many cancers, and it is an important mechanism of cell growth, development, metabolism, and regeneration (Evan and Vousden, 2001; Icard et al., 2019), which is closely related to the degree of tumor differentiation, invasion area, vascular invasion, and lymphatic metastasis (Machlowska et al., 2018).

Based on these DE ferroptosis-related lncRNAs, we have constructed a prognostic model for gastric cancer. The AUC values for the training and test groups reached 0.751 and 0.747, respectively, in our study. In previous studies, other evaluation models have also been constructed to evaluate the prognosis of gastric cancer patients. In the study of Jiang et al., a prognostic model for gastric cancer was constructed based on ferroptosis-related genes. In their study, the AUC values were 0.654 at 1 year, 0.657 at 3 years, and 0.733 at 5 years (Jiang et al., 2021), which were lower than those of our model. The work of Chen et al. (2021) has built a prognostic signature model based on 10 hypoxia-related lncRNAs; the AUCs of this model were 0.703 and 0.734 in the test group and the combined group, respectively (Chen et al., 2020). Using autophagy-related genes, the work of Chen et al. (2021) has built a prognostic model based on five genes; the AUC for this model was 0.736 (Chen et al., 2021). In addition, some other prognostic models were constructed in recent years; however, the AUC values for these models were lower than 0.7 (Liu et al., 2018; Hu et al., 2019; Yu et al., 2019; Qi et al., 2020). This result indicates that the prognostic model based on 17 ferroptosis-related lncRNAs constructed in our study might improve the prognostic power for gastric cancer. Our study still has some limitations. We constructed a ferroptosis-related lncRNA gastric cancer prognostic model just through statistical analysis. More clinical validation would need to be performed in further studies to improve our prognosis prediction model. Also, in further studies, we will analyze the regulatory functions of the 17 ferroptosis-related lncRNA signatures. All in all, the prognostic power of our model was better than that of other published models. Our study might improve the prognosis prediction for gastric cancer patients.

## Data Availability Statement

The datasets presented in this study can be found in online repositories. The names of the repository/repositories and accession number(s) can be found in the article/Supplementary Material.

## Author Contributions

ZX provided the idea. JP and XZ performed the data statistics. ZX and XZ prepared the manuscript. XF and ZX reviewed and modified the manuscript. All authors have reviewed the final version of the manuscript.

## Conflict of Interest

The authors declare that the research was conducted in the absence of any commercial or financial relationships that could be construed as a potential conflict of interest.

## Publisher’s Note

All claims expressed in this article are solely those of the authors and do not necessarily represent those of their affiliated organizations, or those of the publisher, the editors and the reviewers. Any product that may be evaluated in this article, or claim that may be made by its manufacturer, is not guaranteed or endorsed by the publisher.

## Footnotes

1
https://cancergenome.nih.gov/

2
https://www.gencodegenes.org/

3
http://www.zhounan.org/ferrdb/

4
http://www.gsea-msigdb.org/gsea/index.jsp

5
https://www.string-db.org/

## Supplementary Material

Supplementary Figure 1 **(A)** Forest plot for the prognostic values of the ferroptosis related lncRNA signatures through uni-variate COX regression analysis. **(B–C)** And LASSO regression was performed, calculating the coefficients **(B)** and minimum criteria **(C)**.

Supplementary Figure 2 GO annotation analysis for screened 17 ferroptosis related lncRNA signatures.

Supplementary Table 1 Ferroptosis related download from FerrDb database.

Supplementary Table 2 Differentially expressed genes among gastric cancer tissues.

Supplementary Table 3 Differentially expressed lncRNAs among gastric cancer tissues.

Supplementary Table 4 Differentially expressed ferroptosis related genes.

Supplementary Table 5 Intermolecular co-expression relationship details for the ferroptosis related lncRNA co-expression network.

Supplementary Table 6 PPI relationship details.

Supplementary Table 7 The KEGG enrichment analysis details.

Supplementary Table 8 GO annotation analysis details.
