# Supplementary figures and images for: Construction on of a Ferroptosis-Related lncRNA-Based Model to Improve the Prognostic Evaluation of Gastric Cancer Patients Based on Bioinformatics
Source: Front Genet. 2021 Aug 23;12:739470. doi: 10.3389/fgene.2021.739470 (PMC8419360; doi:10.3389/fgene.2021.739470)

[illegible]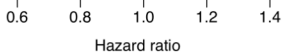

Partial Likelihood Deviance

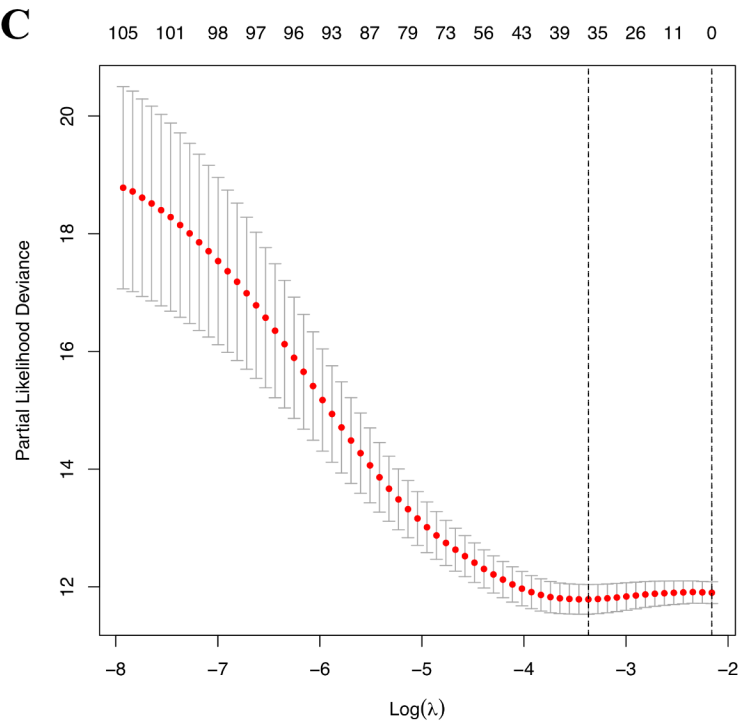

Supplement: Supplementary Figure 1 — (A) Forest plot for the prognostic values of the ferroptosis related lncRNA signatures through uni-variate COX regression analysis. (B–C) And LASSO regression was performed, calculating the coefficients (B) and minimum criteria (C). [file Image_1.PDF]

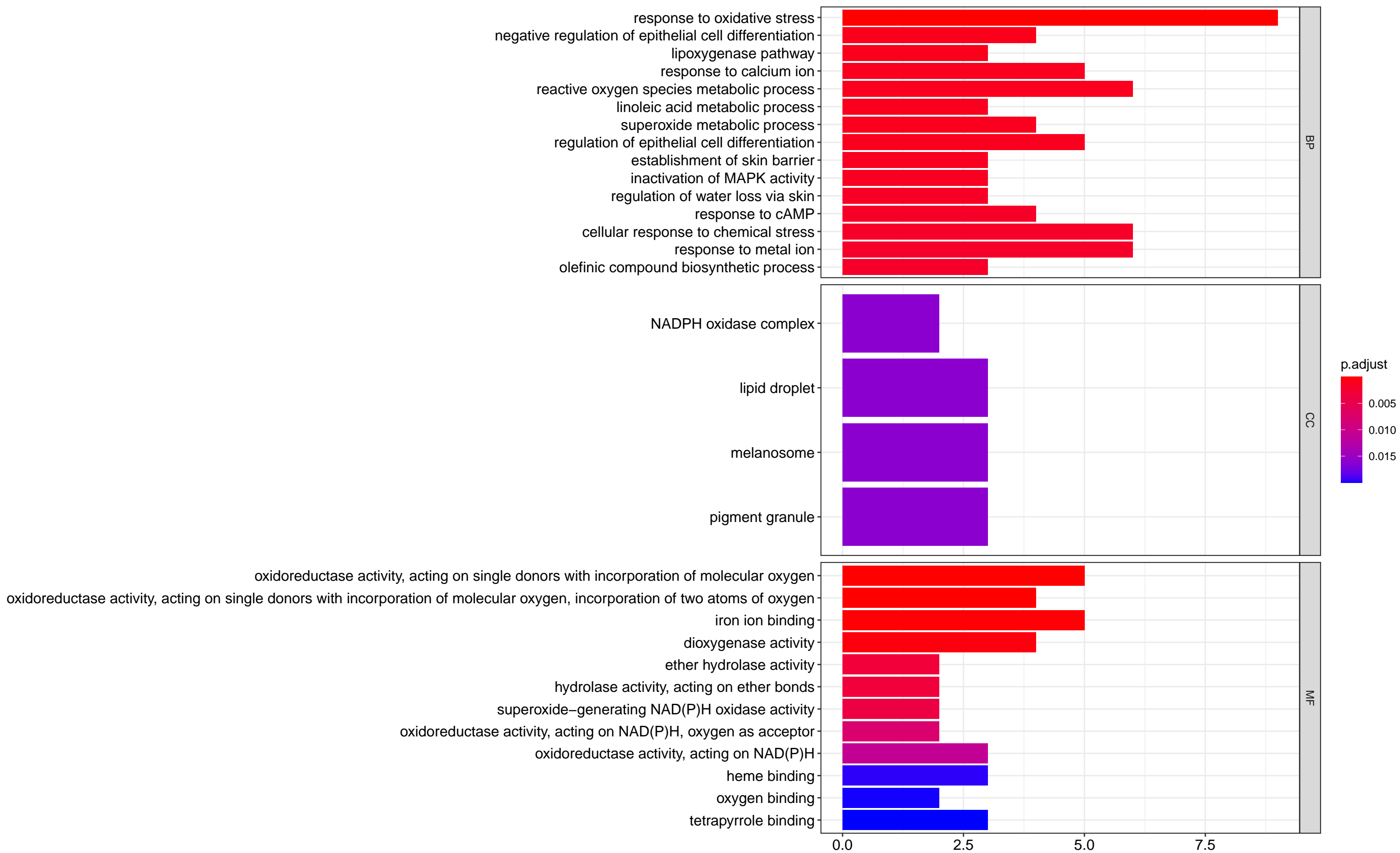

Supplement: Supplementary Figure 2 — GO annotation analysis for screened 17 ferroptosis related lncRNA signatures. [file Image_2.PDF]
